# Comparison of Hospital-Wide Code Rates and Mortality Before and After the Implementation of a Rapid Response Team

**DOI:** 10.7759/cureus.2043

**Published:** 2018-01-09

**Authors:** Muhammad Yousaf, Sheher Bano, Muhammad Attaur-Rehman, Chaudhary Muhammad Junaid Nazar, Aayesha Qadeer, Salma Khudaidad, Syed Waqar Hussain

**Affiliations:** 1 Critical Care Medicine, Shifa International Hospital, Islamabad, Pakistan; 2 Department of Nephrology and Renal Transplantation, Shifa International Hospital, Islamabad, Pakistan; 3 Department of Pulmonology & Critical Care Medicine, Shifa International Hospital, Islamabad, Pakistan; 4 Obstetrics and Gynecology, Bolan Medical College; 5 KRL Hospital Islamabad

**Keywords:** rapid response team, code blue, mortality

## Abstract

Objective

To compare hospital-wide code rates and mortality before and after the implementation of a rapid response team (RRT).

Study design

A prospective cohort design with historical controls.

Place of study

This study was conducted at Shifa International Hospital, Islamabad, from January 21, 2016, to January 20, 2017.

Materials and methods

The triggers for the rapid response team (RRT) were displayed on each floor. The in-house staff was trained on when and how to activate the rapid response team (RRT). Data were collected on a specified data collection form. Mortality and hospital-wide code blue rates were calculated and compared with those from one year before the implementation of the rapid response team (RRT) (i.e., from January 21, 2015, to January 20, 2016).

Results

The total number of admissions during the study period was 40,177. In total, 796 RRTs were activated with a rate of activation of 19.81 per 1000 admissions. The most common activator for RRTs was an altered level of consciousness (24.12%), followed by tachycardia (19.22%), and tachypnea (14.45%). The total number of admissions one year before the implementation of the RRT was 39,460. The total number of mortality events before the implementation of the RRT was 1470 (3.725%) and after the implementation of the RRT was 1529 (3.805%), which was not significantly different (P = .576). The total number of code blues before the implementation of the RRT was 146 (0.369%) and after the implementation of RRT was 148 (0.368%), which was not significantly different (P = .929).

Conclusion

In this large single-institution study, rapid response team implementation was not associated with significant reductions in either hospital-wide code blue or mortality.

## Introduction

In-hospital cardiac arrests continue to have a mortality rate of 80% despite advances in health care [1]. These deadly in-hospital cardiac arrests are preceded by warning signs, such as the derangements of vital signs [2], and the early detection of these warning signs may provide an opportunity to prevent cardiac arrest and its attendant mortality. Based on this, the use of a rapid response team (RRT) has been recommended as a means of reduction of in-hospital mortality [3-4]. An RRT is typically a multidisciplinary team, consisting of a physician, critical care nurse, and respiratory therapist charged with the prompt evaluation, triage, and treatment of patients with signs of clinical deterioration not treated in the intensive care unit (ICU) [5]. The team is triggered by in-house staff when certain predetermined signs of clinical deterioration are observed and can order critical laboratory and imaging studies and medications, transfer the patient to higher levels of monitoring and care, and discuss end-of-life care with the patients and their families independent of the primary physician [6].

Whether the implementation of RRTs is effective in decreasing the hospital-wide code rates and hospital mortality is unclear. Previously conducted studies have shown conflicting results. A few studies have shown that the implementation of an RRT has reduced the out of ICU cardiopulmonary arrests [7-9]. One large study [10] that included 73,000 patients in the post-intervention group, as well as a few small, short-term studies [7,9], have found a significant reduction in hospital-wide mortality after RRT implementation. On the other hand, several other studies have failed to demonstrate a significant reduction in hospital-wide mortality after RRT implementation [11-12], including three of the four largest studies evaluating hospital-wide mortality, with between 25,000 and 68,000 patients in the post-intervention group [8,11-12]. Moreover, a meta-analysis of 17 studies, including more than 400,000 patients, failed to find a reduction in overall hospital-wide mortality or adult mortality, although a significant reduction in pediatric mortality was observed [13].

To address these conflicting results of previous studies, we have conducted our study. Also, we wanted to know the common triggers for RRT activation in our hospital and the common interventions that the RRT team does after it is activated.

## Materials and methods

The study design was a prospective cohort with historical controls. The study was carried out at Shifa International Hospital, Islamabad, a tertiary care hospital having surgical and medical subspecialties along with liver and bone marrow transplant departments, from January 21, 2016, to January 20, 2017. Prior to the implementation of the RRT, teaching and training sessions were carried out with all those who had to participate in the RRT system.

Signs and symptoms were displayed in the reception area of every floor, and the floor doctors and nursing staff were trained to activate the rapid response team if they notice any one of these signs and symptoms in any patient. RRT activation occurred via dedicated pagers through the operator with a single emergency telephone number. The team was led by a critical care fellow and included an ICU nurse and respiratory therapist having all the necessary emergency equipment, such as a bag mask, a glucometer, and an endotracheal tube. When activated, the RRT was supposed to be present on the required floor within 10 minutes and was independent in its decision-making (e.g., advising laboratory and radiology investigations, prescribing medications, shifting the patient to a more intensively monitored area, discussing and changing the code status of the patient, continuing to manage and monitor the patient at the same place, etc.)

Data on hospital-wide mortality, code blue, and demographic characteristics were collected prospectively in the hospital core patient database and retrieved for the study. The data on the primary reason for rapid response team activation, rapid response team intervention, and patient disposition after rapid response team evaluation were prospectively collected on a data collection form by the RRT team. All patients admitted to the hospital during the duration of the study, irrespective of their age and gender, were included. Data on code blues, including location, type of arrest, and initial rhythm were similarly collected. Patients who were in procedure rooms when the RRT or code blue team was activated for them were excluded because the procedure room environment is different from the general ward environment. The hospital-wide mortality and code blue data one year before the implementation of the RRT system, from January 20, 2015, to January 20, 2016, were retrieved from the in-hospital core patient database and were compared with the mortality and code blue data collected one year after the implementation of the RRT system (i.e., from January 21, 2016, to January 21, 2017).

## Results

**Figure 1:**
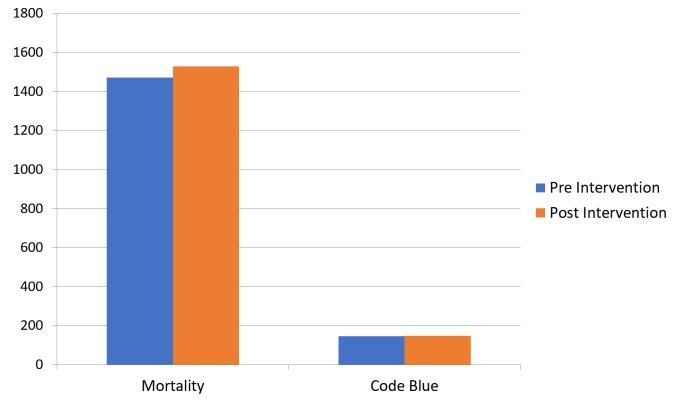
Mortality and code blue before and after the implementation of the rapid response team

The total number of admission before the implementation of the RRT was 39,460 and after the implementation, it was 40,177. There was no statistically significant difference in hospital-wide mortality and code blue rates before and after the implementation of the RRT system. The total number of mortalities before the implementation of RRT was 1470 (3.725%) and after the implementation of RRT, it was 1529 (3.805%) with a P value of 0.576. The total number of code blues before the implementation of RRT was 146 (0.369%), and after the RRT implementation, the total was 148 (0.368%) with a P value of 0.929 (Table 1 and Figure 1) 

**Table 1:** Summary of study outcomes before and after the implementation of the rapid response team Abbreviations: SD, standard deviation.

Outcome	Pre-intervention	Post-intervention	P value
Admission	39460	40177	
Mortality	1470 (3.725%)	1529 (3.805%)	0.576
Code Blue	146 (0.369%)	148 (0.368%)	0.929
Mean Age, Years (SD)	42.9 (18.3)	44 (18.2)	
Females (Percentage)	18388 (46.6%)	18481 (46%)	
Case Mix Index	1.89	1.95	

In total, 796 RRTs were activated. The most common reason for RRT activation was an alteration in the level of consciousness (192 events; 24.12%) followed by a heart rate of > 140 (153 events; 19.22%) and tachypnea (115 events; 14.45%). The other reasons for RRT activation were hypotension, desaturation, chest pain, uncontrolled bleeding, staff/family concerns, hypertension, bradycardia, seizures, and oliguria (Table 2).

**Table 2:** Reasons for rapid response team activation Abbreviations: bpm, beats per minute; LOC, level of consciousness; pm, (breaths) per minute; RRT, rapid response team; SBP, systolic blood pressure.

Indications for RRT activation	Number	Percentage
Alteration in LOC	192	24.12
Heart rate > 140 bpm	153	19.22
Respiratory rate > 28 pm	115	14.45
SBP < 80 mmHg	92	11.56
O_2_ saturation < 85%	76	9.55
Chest pain	67	8.42
Uncontrolled bleeding	40	5.03
Staff/family concerns	35	4.40
SBP > 180 mmHg	12	1.51
Heart rate < 40 bpm	8	1.01
Seizures	4	0.50
Urine output < 20 ml/hr	2	0.25

**Figure 2:**
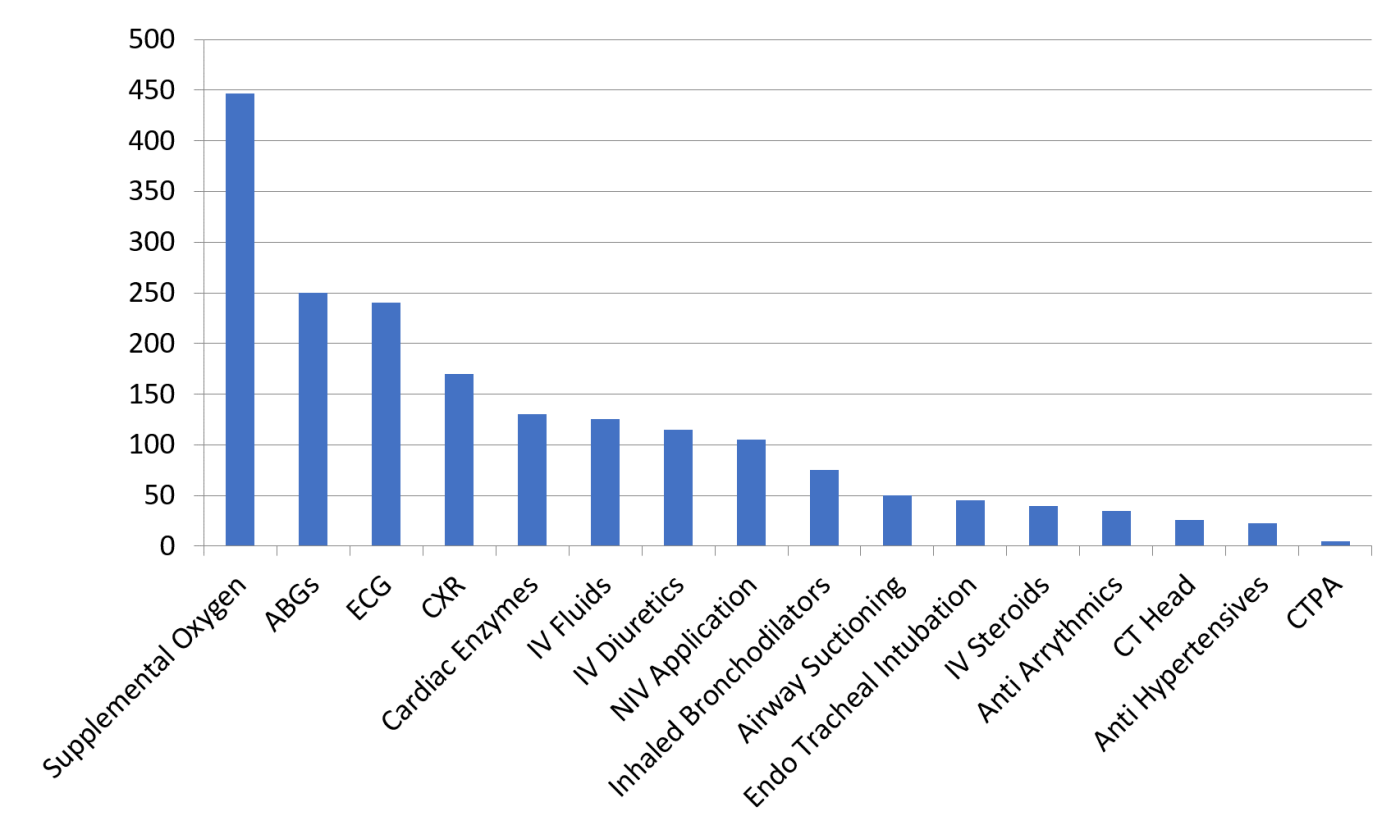
Interventions taken by the rapid response team Abbreviations: ABGs, arterial blood gases; CT, computed tomography; CTPA, computed tomography pulmonary angiography; CXR, chest x-ray; ECG, electrocardiography; IV, intravenous; NIV, non-invasive ventilator

The most common intervention taken by the RRT was supplemental oxygen (447 events; 58.13%) followed by arterial blood gas (250 events; 32.51%) and electrocardiogram (240 events; 31.21%). The other interventions taken by RRT were, in descending order of frequency, chest x-ray, cardiac enzymes, intravenous fluids, intravenous diuretics, non-invasive ventilator application, inhaled bronchodilators, airway suctioning, endotracheal intubation, intravenous steroids, anti-arrhythmic, computed tomography (CT) of the head, antihypertensives, and CT pulmonary angiogram (Figure 2).

The disposition of patients after RRT activation is shown in Table 3.

**Table 3:** Disposition of patients after rapid response team activation Abbreviations: CCU, coronary care unit; DNR, do not resuscitate; HDU, high dependency unit; ICU, intensive care unit; RRT, rapid response team; S.No., site number.

S.No.	Disposition Place	Number	Percentage
1.	HDU	366	45.98
2.	REMAINED ON FLOOR	207	26.01
3.	ICU	107	13.44
4.	CCU	72	9.05
5.	CODE STATUS CHANGED TO DNR	40	5.03
6.	DEATH DURING RRT	4	0.50

The 40 patients whose code status was changed to do not resuscitate remained on the floor and were excluded from the study. Of the remaining 756 patients, 636 were discharged home safely, and 120 expired during the hospital stay; the expiry site of these patients by number and percentage is shown in Table 4

**Table 4:** Site of expiry of patients during hospital stay Abbreviations: ICU, intensive care unit; RRT, rapid response team; S.No., site number.

S.No.	Place of Death	Number	Percentage
1.	Within ICU	50	41.67
2.	Outside ICU	66	55.00
3.	During RRT	4	3.33

## Discussion

Our study compared the hospital-wide mortality and code blue rates one year before and after the implementation of the rapid response team. In addition, we evaluated the various characteristics of our rapid response team, such as the triggers of activation, the interventions taken by the rapid response team, and the disposition of the patients after rapid response team (RRT) activation. Our study included more than 40,000 patients. We have found no difference in hospital-wide mortality and code blue rates before and after the implementation of RRT. This finding is in accordance with the work of other investigators, including a large multicenter randomized controlled trial, the Medical Early Response Intervention and Therapy (MERIT) study, which did not show any improvement in code blue rates after RRT implementation [11] and a meta-analysis of 18 studies that concluded that robust evidence of the effectiveness of rapid response teams in reducing hospital mortality is lacking [13]. Although some prior studies and a meta-analysis have shown that the implementation of the RRT has decreased out-of-ICU code blue rates [9,13], the extent to which this decrease is due to a reversal in physiological decline after RRT intervention or because of the transfer of the patients to the ICU by the RRT team, thus removing a potential code blue event from the study outcome because within-ICU code blues are not announced, is not known.

The code status of 40 patients out of a total of 796 patients (5.20%) was changed to the ‘do not resuscitate’ status. It may be a contribution of the RRT directing hospital resources and personnel to patients who are more likely to benefit and referring those patients who need end-of-life care to the palliative care department but how much this is cost-effective was not determined. Our study should be interpreted in the context of following limitations. Though it had more than 40,000 patients in the post-intervention period, it was a single institute study and included both adult and pediatric populations. Outcomes may have been different if both populations had been studied separately. All those who were involved in the activation of the RRT were trained before the implementation of the RRT on those signs and symptoms that should trigger the activation of the RRT. These were displayed in the reception area of every floor, yet, a delay in the activation of the RRT may be a factor in the lack of improvement in hospital-wide mortality and code blue rates after the implementation of the RRT. Our study was a prospective cohort study with historical controls; the two cohorts may not be identical. This is a common problem with all such studies. However, there was no epidemic in the post-implementation. As the post-implementation period was one year and was compared with the population from one year before the implementation, seasonal variation was not an issue. The hospital-wide efforts to improve the quality of patient care would have been expected to decrease the hospital-wide mortality rate and in-hospital code blue rate. However, in our study, there was no difference in mortality rate and code blue rate before and after the implementation of the RRT; therefore, the quality improvement effort was not a confounding factor.

## Conclusions

There was no significant difference in the hospital-wide mortality rate and the in-hospital code blue rate before and after the implementation of a rapid response team in our tertiary care hospital. As our study failed to show any improvement in hospital-wide mortality rate and code blue rate after the implementation of the rapid response team, it is recommended that multicenter studies with a sufficiently long follow-up be conducted before suggesting the implementation of a rapid response team in tertiary care hospitals across the country.
